# A Review of Research on the Effect of Temperature on the Properties of Polyurethane Foams

**DOI:** 10.3390/polym14214586

**Published:** 2022-10-28

**Authors:** Juan Wang, Chenxiao Zhang, Yu Deng, Peng Zhang

**Affiliations:** 1 Yellow River Laboratory, Zhengzhou University, Zhengzhou 450001, China; 2 School of Water Conservancy Engineering, Zhengzhou University, Zhengzhou 450001, China; 3 Yellow River Institute of Hydraulic Research, Zhengzhou 450003, China

**Keywords:** polymer, rigid polyurethane foams, temperature, mechanical properties

## Abstract

Temperature is one of the main factors affecting the properties of polyurethane foams, and there are large differences in the mechanical properties of polyurethane foams at different temperatures. To understand the effect of temperature on the mechanical properties of polyurethane foams and to provide a theoretical basis for the application of polyurethane foams in extreme environments, this paper systematically describes the research on the effect of mold temperature, raw material temperature, and environmental temperature on the microstructure and mechanical properties of polyurethane foams in the formation and service stages of rigid polyurethane foams by domestic and foreign scholars, and summarizes the effect of temperature on the mechanical properties of polyurethane foams and the mechanism of action. A review of the literature shows that the effect of different temperatures on the mechanical properties of polyurethane foams can be summarized. The literature review shows that there are certain changes in the foaming process, pore structure, and mechanical properties of polyurethane foams at different temperatures, and the increase in temperature generally leads to the increase in pore size, decrease in density, and decrease in mechanical properties of polyurethane foams.

## 1. Introduction

Polymeric materials are mainly divided into three categories: plastics, rubber, and fibers, among which plastics are the most produced, accounting for about 70–75% of the total, and are now used on a large scale in agriculture [1], transportation [2], construction engineering, medical treatment [3] and defense construction [4]. Polyurethane foams material is an important synthetic material made by the reaction of polyol and isocyanate, which has the characteristics of large adjustable formulation, various types of products, excellent mechanical properties, and simple processing and molding methods [5,6]. Depending on the design of the formulation, the desired product can be prepared. There are many varieties of polyurethane foams to meet the needs of different fields, and the types of products include foaming materials, elastomers, adhesives, paving floors, waterproof materials, fiber synthetic leather, water-based coatings, and many other types. Polyurethane foam has low density, heat insulation, shock absorption and cushioning, sound absorption and noise prevention, good mechanical properties, etc. All the above advantages make polyurethane foams one of the indispensable polymer materials in the world today, which is widely used in oil pipelines, transportation, construction, electronic household appliance, medical equipment, sports equipment [7], etc.

Polyurethane foam is the product form that accounts for the largest proportion of polyurethane foams synthetic plastics and is a fast-growing species in the plastics industry [8,9]. It is defined as a porous polyurethane foams synthetic material consisting of polyurethane foams resin foam meridians and a large number of microporous cells, called “Polyurethane Foam (PUF)” in English [10]. From the definition, it is clear that the main characteristic is porosity, which, combined with the excellent mechanical properties of polyurethane foam materials, makes polyurethane foams have low density, high specific strength, and a wide range of adjustable properties [11].

According to the degree of softness and hardness of polyurethane foam, it can be classified as flexible foam, semi-rigid, and rigid foam. Among them, rigid polyurethane foam (RPUF) is a polyurethane foam that does not undergo significant deformation under a certain load and cannot return to its initial state when deformation occurs under excessive load. In recent years, RPUF materials have been widely used in water conservancy [12], civil engineering [13], transportation [14,15], and mining [16,17] because of the advantages of no maintenance, fast reaction molding, lightweight, and good mechanical properties, etc. Its main role in water conservancy projects is to replace some cement-based materials with polyurethane foam materials as grouting materials for foundation treatment [18] and seepage control and repair [19,20], etc. As early as the early 20th century, academician Wang Fuming of Zhengzhou University developed a complete set of technologies for the rapid inspection and repair of highways based on nondestructive testing and two-component foamed polyurethane foams grouting in combination with the structural characteristics and typical diseases of highways in China [21,22] and was widely used. In recent years, it has been widely used in water conservancy engineering grouting [23,24]. The compression [25,26], tensile [27,28], and shear properties [29] as well as the strain rate effect [27] and fracture properties [30] of polyurethane foams at different densities have been studied, laying the foundation for the study of the mechanical properties of polyurethane foams. The main factors that affect the mechanical properties of polyurethane foams are density, temperature, humidity, and strain rate. As the application field of polyurethane foam materials is more and more extensive, their service conditions are more and more complicated, and some projects need to use polyurethane foam materials under cold or hot conditions [31,32]; therefore, to meet the demand for the temperature of polyurethane foams in actual engineering, the research on temperature-influenced mechanical properties of polyurethane foams has also involved more and more scholars in recent years. In this paper, the research on the influence of temperature on the mechanical properties of polyurethane foams by domestic and foreign scholars is reviewed, and the research on the influence of temperature on the properties of polyurethane foams in the forming stage and the service stage is reviewed, which provides a theoretical basis for the polymer materials serving in extreme environments in the future.

## 2. Influence of Temperature on Polyurethane Foam Properties during the Molding Stage

The mechanical properties of polyurethane depend mainly on the cell structure, including cell size [33,34,35], cell content, anisotropy of the cell structure [36,37,38], closed (cell content), and wall thickness of the cell [39]. The formation stages of polyurethane foams include four phases cell nucleation, cell growth, microphase separation, and polymerization [40]. In the process of foaming to molding, ambient temperature, raw material ratio, mold temperature, material temperature, and curing time affect the foaming rate and the final cell structure morphology of polyurethane foams. The level of material and ambient temperature during the foaming to molding process directly affects the quality of the foam products, and thus the performance of polyurethane foams polyurethane foams to varying degrees.

### 2.1. Effect of Mold Temperature on Polyurethane Foams

Mold temperature is the key factor of foam molding, as the name implies; the mold of polyurethane foam material is processed to different temperatures and then the foam material is put into the mold for foaming. Mold temperature not only affects the speed of reaction heat removal, but also the molding time, and affects density, cell structure, and dimensional stability.

#### 2.1.1. Influence on Cell Structure

Mold temperature affects the cell structure of polyurethane foams, and for the microstructure of polyurethane foams cast at different mold temperatures, Mohan et al. [41] carried out a microscopic experiment for observation, and the results showed that polyurethane foams treated at low temperatures produced anisotropic cell content of uniform size toward the upward direction. The limited passage between the cells through the cell walls resulted in stiffness. However, at an elevated temperature, the polyurethane foams produced more isotropic spherical cells with a larger change in size and no specific orientation, with significantly thicker cell walls. Abdul-Rani [42], on the other hand, explored the effect of polyurethane foams at different mold temperatures on the surface texture of the foamed polyurethane foams, which varied in roughness at different mold temperatures, observing that mold temperatures at 40 °C and 60 °C had the smoothest skins. In addition, mold temperature can affect the density as well as the density gradient of polyurethane foam materials [43]. Researcher Mohan [41] foamed polyurethane foams materials at different mold temperatures and observed their density after foaming, finding that as the temperature increased, the foaming chemicals could not fill the cylinder due to the increase in viscosity during foaming. Voids are created where the foam cannot be filled, and the polyurethane foams density decreases to varying degrees. Furthermore, studies by DaciaJackovich [44], Harbron [45], and Yeon [46] showed that increasing the mold temperature can effectively reduce the average density gradient, the density in the growth direction, and the uniformity of the cell (cells) of the foam. As the mold temperature increased, the average polyurethane foams cell size became larger, and the dimensional stability improved significantly. Unfortunately, only qualitative analyses are available on the effect of mold temperature on the microscopic cell structure of polyurethane foam materials, and statistics on the changes in its cell size or porosity are temporarily lacking.

#### 2.1.2. Effect on Mechanical Strength

The change in mold temperature caused the change in cell structure and therefore the change in mechanical properties of polyurethane foams. Scholars Mohan [41] and Dacia Jackovich [44] measured the mechanical properties of polyurethane foamed successfully at different mold temperatures. The general results show that with the increase in mold temperature, the mechanical properties of polyurethane foams materials such as compression, tensile, shear strength and elastic modulus have a significant decreasing trend. Furthermore, Xiang [47] investigated the effect of mold temperature (40 °C, 60 °C, 80 °C, 100 °C) on the mechanical response of injection molded polyurethane foams materials at different ambient temperatures and molecular structures, and the stress–strain curves obtained indicated that the increase in mold temperature led to a decrease in tensile strength, as well as tensile modulus and an increase in tensile yield strain. Some scholars have come to different conclusions. Wang [48] selected four process factors: mass filling factor, mold temperature, material temperature, and stirring speed, to conduct a 4-factor, 3-level orthogonal test on the foaming process of RPUFs, and discussed the effects of process conditions on the structure and properties of RPUFcells by analyzing the experimental data. The results show that increasing the mold temperature can effectively improve the overall density in the growth direction and the uniformity of the foam cell.

**Figure 1:**
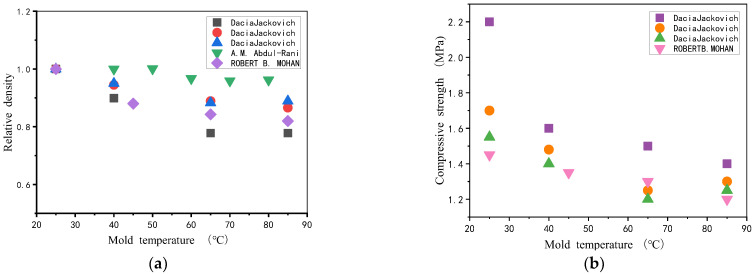
(**a**) Variation of polyurethane foams density at different mold temperatures; (**b**) Variation of polyurethane foams compression strength at different mold temperatures.

Figure 1 collate the density and compression strength of polyurethane foam materials at different mold temperatures. Because of the large density span involved in the data, to more intuitively show the overall change trend of the data, this paper adopts the normalized way to process the data of temperature-influenced polymer density. It can be seen from the figure that with the increase in the mold temperature, the density and compressive strength of the polyurethane foams both show a downward trend, and the change range of the temperature above 65 °C slows down.

### 2.2. The Effect of Ambient Temperature on Polyurethane Foams

The foam forming of polymeric materials is also related to the ambient temperature they are exposed to [49], while Wang [50] investigated the effect of ambient temperature on the homogeneous cell forming of foams, and studied the effect of ambient temperature on cell size uniformity under the same formulation system according to the change in atmospheric environment (the temperature of raw materials is consistent with the temperature of foaming environment). The experimental results show that the foam rise time decreases sharply with the increase in temperature. The effect of ambient temperature and blowing agent content on the foaming properties of polyurethane foams was studied by Kim [51]. In this study, the foaming behavior of semi-rigid polyurethane foams was observed by using two main processing variables, such as ambient temperature and blowing agent content, for the cup-shaped foam test. The lower the ambient temperature, the denser the foaming and the lower the foaming height, and the higher the ambient temperature, the more complete the foaming and the larger the cell size. All these studies indicate that when the ambient temperature increases, the material temperature and mold temperature increase accordingly, which increases the reactivity of polymer molecules and makes polymer molecule collisions intensify. While the temperature increases, the heat dissipation during the reaction of the mixture is small, and the temperature during the reaction increases relatively, which increases the polymerization reaction rate and the foaming rate and makes the polymer produce more microporous structures or the volume of microporous structures becomes larger [52].

### 2.3. Influence of Initial Mixture Temperature on Polyurethane Foams

**Figure 2:**
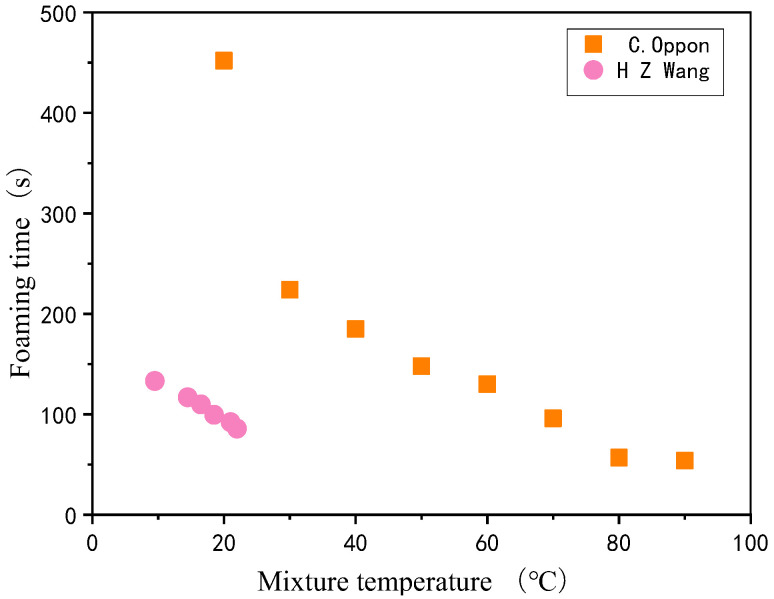
Variation of polyurethane foams foaming time at different material temperatures.

**Figure 3:**
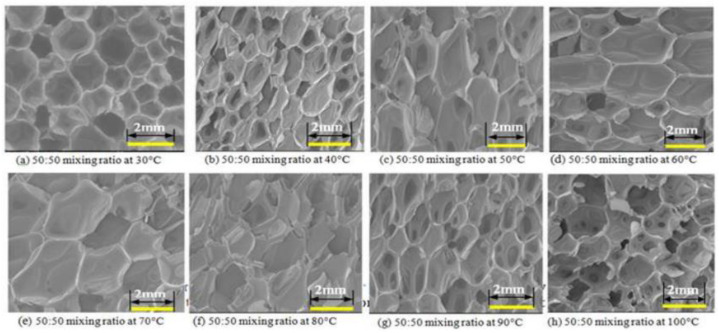
SEM images of PU foams produced at preheating temperatures of 30–100 °C. Reprinted from [55].

The starting conditions of the reactants not only determine the cell structure of the foam material [53] but also affect the polyurethane foams foaming time. Wang [54] et al. studied the foaming process parameters of two different polyurethane rigid foam composites formulations by linearly fitting the measured reaction times to the corresponding feed temperatures. The results are shown in Figure 2, where a linear relationship between the initial mixture temperature and several foaming time parameters, with an increase in the material temperature shortening the foaming time. Oppon [55] preheated the polyurethane foams initial mixture at several preheating temperatures from 20 °C to 100 °C at 10 °C per step, and SEM microscopic experimental observations, as well as mechanical property tests, were performed after the foaming was completed. As shown in the Figure 3, the porosity increased from 33.3% at 20 °C to 45.2% at 60 °C and decreased to 37.3% at the specified 100 °C. The porosity of the polyurethane foams material gradually increased with the increase in preheating temperature and the corresponding density decreased. The results of mechanical properties tests showed that both tensile strength and modulus of polyurethane foams materials gradually decreased with the increase in preheating temperature. Özdemir [56] used different proportions of the initial mixture and initial mixture temperature (20–45 °C) for polyurethane foams foaming and the results showed that a higher initial mixture temperature can significantly reduce polyurethane foams filling time and improve foaming uniformity.

In summary, both mold temperature, ambient temperature, and raw material temperature affect the chemical reaction in the polyurethane foams foaming process. In the formation stage, polyurethane foams generally undergo two processes: gelling reaction, foaming, and curing reaction [57]; the chemical reaction process is shown in the following formula.

(1) $$\begin{array}{l} \mathrm{OCN}\underset{\mathrm{isocyanate}}{-R-}\mathrm{NCO}+\mathrm{HO}-\underset{\mathrm{polyol}}{R^{\prime }}-OH\to \\ -\left[O-R^{\prime }-O-\underset{\mathrm{polyurethane}}{\mathrm{CONH}}-R-NHCO\right]-\underset{n}{} \end{array}$$

(2) $$\underset{\mathrm{isocyanate}}{R-\mathrm{NCO}}+\underset{\mathrm{water}}{H_{2}}O\to R-\underset{\mathrm{carbami}\;\mathrm{cacid}}{\mathrm{NHCOOH}}$$

(3) $$\underset{\mathrm{isocyanate}}{R-\mathrm{NCO}}+\underset{\mathrm{water}}{H_{2}}O\to R-\underset{\mathrm{carbami}\;\mathrm{cacid}}{\mathrm{NHCOOH}}$$

(4) $$\underset{\mathrm{isocyanate}}{R-\mathrm{NCO}}+\underset{\mathrm{amine}}{R-{\mathrm{NH}}_{2}}\to R-\mathrm{NH}-\underset{\mathrm{urea}}{\mathrm{CO}}-\mathrm{NH}-R$$

Polyurethane foam materials are formed by the gradual polymerization of isocyanates, polyesters, polyethers, polyolefins, and isocyanate end groups of diols or diamine compounds and prepolyurethane foams [58]. The higher the temperature during the casting process, the higher the activity of the reaction material and the more the –NCO bond of polyisocyanate and the -OH bond of polyether polyol can fully react, while at a lower reaction temperature, Insufficient reaction between –NCO bond of polyisocyanate and –OH bond of polyether polyol will inevitably lead to low strength of prepared polyurethane foam [46]. At the beginning of the reaction, the chain expansion reaction gives off a large amount of heat, which leads to a rapid increase in the temperature of the material system. The viscosity of the liquid first decreases and then increases. As the reaction proceeds, more and more carbon dioxide is produced. The carbon dioxide nucleates in the liquid to form tiny cells, and then rises [59].

**Figure 4:**
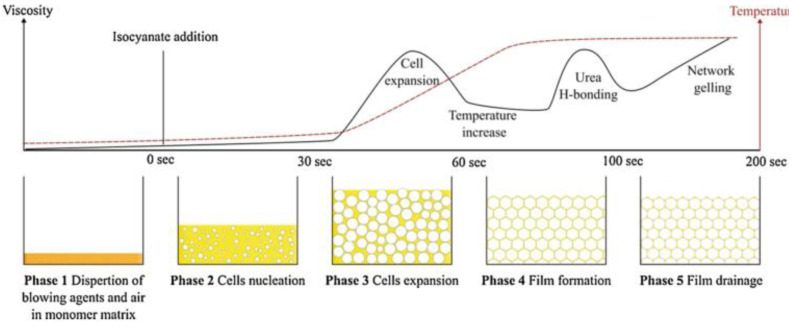
Growth of cells during polyurethane foams formation. Reprinted from [60] with permission from Elsevier.

As shown in Figure 4, As the viscosity of the system increases and the exothermic rate decreases, the temperature of the reaction system increases slowly accordingly [60]. In the process of cell formation, the mold temperature, raw material temperature, and ambient temperature during foaming directly affect the rate of reaction heat transfer; the lower foaming temperature results in a faster reaction heat transfer, smaller foaming multiples, and smaller cell size and higher density of the finished product. In addition, the lower temperature slows down the polymerization reaction and therefore reduces the reaction rate. This is because the time of diffusion of the gas into the cell is affected and therefore more nucleation sites are formed [61]. In this sense, more nucleation sites allow the formation of smaller cells, allowing the formation of polyurethane foams with an increased density and therefore an impact on the mechanical properties of the polyurethane foams.

## 3. Influence of Temperature on Polyurethane Foam Properties during Service Phase

Since polyurethane materials are widely used in transportation, construction, and grout repair fields, they are subjected to a wide range of environmental temperatures [62,63], and temperature is one of the most critical parameters affecting the service performance of polyurethane foam materials [64]. Changes in environmental temperature also cause changes in their macroscopic properties, so scholars have conducted a series of studies on the various properties of polyurethane foams in service at different temperatures.

### 3.1. Influence of Ambient Temperature on Tensile and Compressive Strength

In the service phase of polyurethane foam materials, scholars at home and abroad have conducted many experimental studies on the effect of ambient temperature on the tensile and compressive strength of polyurethane foams. Vilimek [65], Pang [66], Hu [67] and Song [68] tested the changing pattern of the compressive and tensile strength of RPUF materials with different densities at different temperatures through experiments, and the results showed that the increase in temperature caused the loss of strength and modulus. As the temperature decreased, the modulus and yield strength tended to increase, the elastic strain of the foam increased, but the yield step was shortened, and the plastic deformation decreased. Professor Shi [69], on the other hand, studied the compressive strength of polyurethane foams specimens with different densities under different temperature conditions and obtained the variation law of compressive strength of polyurethane grouting materials with density at different temperatures, and also obtained the volume variation law of polyurethane foam specimens when the temperature changed. Cao [70] conducted axial compression tests on foam-filled GFRP (glass fiber reinforced polyurethane composites) cylinders at 20–110 °C. The effects of temperature, outer wall thickness, and the presence or absence of foam filling on the mechanical properties and energy absorption characteristics of foam-filled GFRP cylinders were investigated. The test results show that the axial compression properties and energy absorption characteristics are influenced by the thickness of the GFRP surface layer, but the temperature plays a decisive role. The ultimate compressive strength of the foam-filled GFRP cylinders decreased significantly with the increase in temperature. Jia [71] experimentally investigated the effect of temperature on the mechanical properties of CFRP (carbon fiber reinforced polyurethane foams composites) composites under static and dynamic three-point bending tests. The results showed that CFRP composites provided stronger flexural strength, maximum deflection, and energy absorption at lower temperatures, while the performance was relatively poor at higher temperatures.

**Figure 5:**
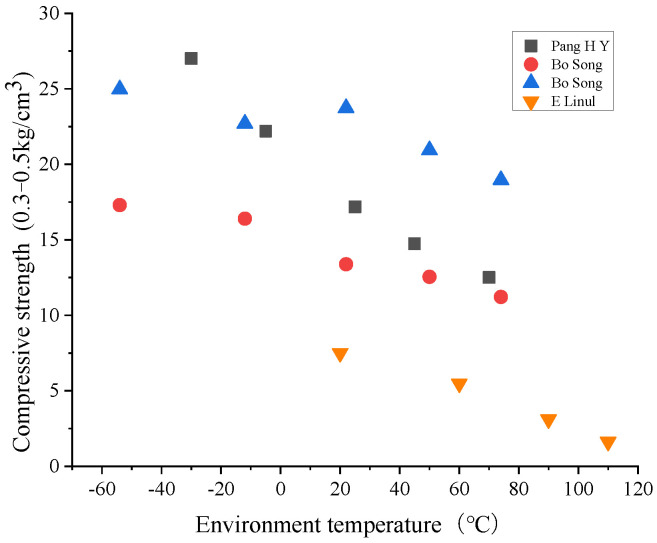
Variation in polyurethane foams compression strength at different ambient temperatures.

Figure 5 collates the trends of compression and strengths of polyurethane foam materials with different densities serving at different ambient temperatures by domestic and international researchers. From the figure, it can be seen that the compressive strength of polyurethane foam materials decreases with the increase in ambient temperature,

To reveal the strength yielding pattern of polyurethane foams with temperature more accurately and intuitively, researchers have developed prediction models for different polyurethane foam materials.

As shown in Table 1, Lee [72] developed a unified strain-rate and temperature-dependent elasto-viscoplastic damage model for polymer foams by improving an existing model, which was used to describe the phenomena of void volume fraction and elastic modulus reduction in polymer foams under uniaxial compression and to evaluate the nonlinear strain-rate and temperature-dependent material behavior of polymer foams. Li [73] obtained the energy storage modulus and loss factor of polymer materials at different temperatures, frequencies, and densities for the dynamic viscoelastic properties of new polymer materials. The factors affecting the viscoelasticity of polymer materials were analyzed and a viscoelastic intrinsic model of polymer materials was developed. Zhang [74] used the three-point bending method. The dynamic viscoelastic parameters of polyurethane foams materials with different densities at different frequencies were measured at −50, −25, 0, 25, and 50 °C. A generalized Maxwell-based dynamic viscoelastic intrinsic model of polyurethane foams grounding materials was proposed, and the correctness and rationality of the intrinsic model were verified. Kopal [75] used an artificial neural network technique for simulating the temperature dependence of the dynamic mechanical properties and viscoelastic behavior of widely developed thermoplastic polyurethane foams over a wide temperature range. Barua [76], on the other hand, developed an SE model successfully used to simulate the stress relaxation behavior of PUFs at different test temperatures and foam densities. Experimental data over a short period were used to predict the relaxation behavior of PUF materials over a long period. Model foam density and temperature terms were combined with improved Gibson–Ashby relations and the Arrhenius equation to develop stress relaxation expressions for polymer foams. Dan [77] investigated the effect of foam density, test velocity, and temperature on densification and efficiency of low and high-density PUF. Reconstructed engineering stress–strain curves were generated using the model. The effects of temperature and test velocity on the onset of densification and absorption of specific strain energy in polyurethane foams were analyzed. Richeton [78] proposed a model conversion to a physically based robust model for predicting the stiffness modulus at various temperatures and frequency/strain rates. Neilsen [79] experimentally investigated the effects of loading path, loading rate, and temperature on polyurethane foam deformation and found that RPUF has a great dependence on strain rate and temperature, and developed a unified creep plastic damage (UCPD) model for RPUF. All the above studies verified the temperature dependence of polyurethane foams by different models.

### 3.2. Influence of Ambient Temperature on Impact Strength

The increase in energy absorbed by polyurethane foams when subjected to dynamic compressive loading is due to the increase in impact resistance and stiffness with decreasing temperature and increasing density [80]. In this regard, many scholars have conducted studies using different methods. E Linul et al. [81] investigated the effects of density, loading rate, material orientation, and temperature on the dynamic compression properties of RPUF materials. The results showed that, as with quasi-static loading, the mechanical properties of the material decreased significantly with increasing temperature at high strain rates. Zhang [80] used dynamic mechanics to analyze the mechanical behavior of polyurethane foams materials at different strain rates (0.001/s to 7000/s) and different temperatures (−40 °C to 25 °C). The results obtained show that the mechanical behavior of polyurethane foams is sensitive to temperature and strain rate. Under dynamic loading conditions, the stress–strain curve at −40 °C changes from “rubbery” to “glassy”. The stress–strain curves at a low strain rate and low temperature can overlap with those at a high strain rate and high temperature; there may be an equivalent relationship between temperature and strain rate. Kim [82] investigated the strain rate and temperature-dependent elasto-viscoplastic behavior of polyurethane foam under static/dynamic compression and low/high temperature, yield stress, plateau height, and densification height decreased. In particular, the slope and densification of the plateau decreased with increasing temperature. Marsavina et al. [83] studied the strain rate and temperature dependent behavior of PUF through experiments. Compression tests were performed on specimens with a density of 200 kg/cm3 at strain rates of 2–360,000 mm/min and temperatures of −60 °C, 23 °C and 80 °C. The experimental results show that the elastic modulus, yield strength and stress drop level of the material increase with the increase in strain rate and the decrease in temperature. In addition, the stress drop region is extended by changing the strain rate and temperature. In addition, due to the influence of temperature on the brittleness of polyurethane foam, the strength ratio at different strain rates also changed.

### 3.3. Influence of Ambient Temperature on Fracture Properties

To understand the mechanical properties of polyurethane foams more fully, it is also important to study the fracture damage mechanism [84,85,86]. The fracture toughness of closed-cell RPUF was studied by E Linul [87] under different loading and temperature conditions. The effect of density and anisotropy on quasi-static and dynamic fracture behavior was also investigated experimentally and the results showed that all polyurethane foam samples showed a significant increase in KIC (fracture toughness) at low temperatures regardless of foam density and loading direction. Park [88] investigated the damage properties and deformation recovery rates of foams at different temperatures and strain rate levels. It was shown that a decrease in temperature had a significant effect on fracture behavior and recovery rate. Jia [89] experimentally studied the type I fracture toughness of the adhesive at low temperature and high loading rate. The load-strain curves of polyurethane foam adhesive at different loading rates (0.5 mm/min, 50 mm/min, 500 mm/min) and different temperatures (room temperature, −20 °C, −40 °C) were obtained. From the experimental results, the type I fracture toughness of polyurethane foams is extremely sensitive to high loading rates and low temperature. As the loading rate increases and the temperature decreases and the type I fracture toughness of this polyurethane foams material decreases significantly.

### 3.4. Effect of High-Temperature Heat Treatment on Polyurethane Foam Properties

During the service life of polyurethane foams, which are susceptible to external aging factors, the macromolecules of RPUF undergo some degree of degradation at high temperatures. Different degrees of degradation eventually degrade the performance of RPUF until failure. Yang [90] et al. investigated the mechanism of thermal degradation of RPUF. They proposed that the degradation reaction of RPUF can be divided into two steps. First, the carbamate bond breaks and releases isocyanate, followed by the major decomposition of the macromolecular backbone, releasing small molecule products such as CO_2_. He [91] subjected RPUF materials to heat treatment at different temperatures. After high-temperature heat treatment, the vibrational frequencies of the isocyanate and amino acid ester groups in RPUF molecules increased, while the RPUF molecular orbital energy gap decreased, and the tensile properties of RPUF decreased with increasing heat treatment temperature. The results of multiscale simulations of the mechanical properties showed that the defects and voids generated in RPUF at high temperatures increased with the increase in heat treatment temperature, which intensified the stress concentration in RPUF and reduced the tensile properties of RPUF. Li et al. [92] studied the dynamic viscoelastic properties of non-aqueous reactive polyurethane foams materials using the dynamic thermo-mechanical analysis (DMA) technique and analyzed the frequency spectrum of non-aqueous reactive polyurethane foams materials, and the results showed that the non-aqueous reactive polyurethane foams materials have good cold and heat resistance, which is related to the mechanical loss of small moving units of polyurethane foam materials. It was tested that the material still has good resistance to low-temperature impact and high-temperature deformation in the range of −60 to 140 °C. Tian [93] studied the microstructure of polyurethane foams materials exposed to 11%, 45%, and 80% relative humidity and 70 °C for 1 and 2 months, and the polyurethane foams chains gradually shortened with increasing humidity and aging time. The shortening of the polyurethane foams chains resulted in a decrease in the glass transition temperature of the soft chain segments and promoted the crystallization of the soft chain segments during prolonged storage of the aged samples at room temperature. Wu [94] investigated the failure mechanism of RPUF at room temperature (RT) and high-temperature vibration conditions through experiments and finite element simulations. The results showed that the tensile strength of polyurethane foams gradually decreased with increasing high-temperature vibration and time, and the vibration damage of polyurethane foams was observed by microscopic experiments mainly due to the presence of microcracks in the cell structure.

Other scholars have proposed that the thermal decomposition of [95] polyurethane began at 170 °C~200 °C, and the carbamate group on the main chain of the polymer broke at the C–O bond, decomposed to form isocyanate and polyol, and then further decomposed into amines, olefins and CO_2_. During the thermal decomposition process part of the diisocyanate products reacted to form diimide. When the temperature reached about 300 °C, the polyurethane was actually decomposed. Starting from 320 °C, diimide is decomposed of isocyanate. For the reaction process, see Formula (5).
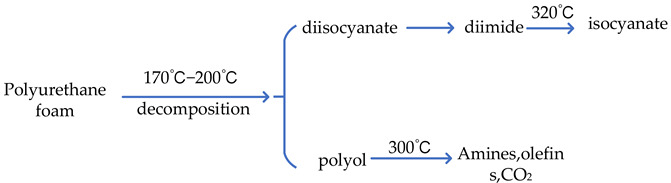
(5)

The decomposition of polyurethane foam is divided into three stages: loss of low-stability organic compounds (bond fission at the weakest link in the chain), oxidative degradation of organic components, and oxidative degradation of residual material [96]. Diurea and urea formate are the weakest links in the flexible polyurethane foam matrix in terms of thermal properties, and at temperatures of 100–200 °C, isocyanate, diurea crosslinking and backbone polyurethane linkages decompose one after another, accompanied by the evaporation of water. The mechanical properties of polyurethane foam degrade sharply at 150 °C. [97] When the temperature rises above 200 °C, the carbamate, as well as the urea groups, start to decompose. The intensity of the absorption band corresponding to the polyol segment decreases, indicating the degradation of the polyol [98]. When the temperature continues to rise to about 300 °C, the hard segment has been completely decomposed. After 340 °C, the carbonyl and ether bonds and other characteristic functional groups are decomposed and each peak in the spectrum is low, indicating the completion of degradation [99].

**Figure 6:**
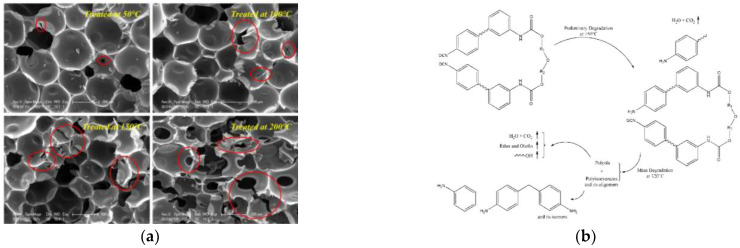
(**a**) Fine-scale structure of untreated RPUF and heat-treated RPUF. Reprinted with permission from [91]; (**b**) Mechanism of RPUF cell failure. Reprinted with permission from [91].

The chemical reactivity of the reactive groups (isocyanate and carbonyl) in the RPUF substrate molecules was further excited as the heat treatment temperature of the polyurethane foam material increased. This led to macromolecular decomposition, which further makes the weak regions in the foam structure more vulnerable when subjected to external loads, causing more stress concentrations and eventually leading to the mechanical failure of the RPUF [100]. The process is shown in Figure 6b.

### 3.5. Properties of Polyurethane at Low Temperature

Rigid polyurethane foam is widely used as thermal insulation material because of its low thermal conductivity and high strength density ratio at low temperatures, such as in refrigerator insulation layers, natural gas pipelines [101], and aerospace materials [102]. As a spraying material, polyurethane foam has all the advantages of more straightforward processing and lower cost. It also shows better mechanical properties at low temperatures than at room temperature and can adapt to low-temperature impact. Therefore, the low-temperature performance of polyurethane foam is also worth exploring. Some scholars characterized the mechanical properties of RPUF at low temperatures by automatic tests. The experimental results show that the RPUF has better compression performance and Young’s modulus at low temperatures with the decrease in temperature [103,104]. The tensile strength and tensile modulus also increase with decreasing temperature and the elongation at break decreases [105,106]. At the same time, by observing the morphology of polyurethane foam when it breaks and the microstructure when it fails at different temperatures, it is found that it shows stronger brittle characteristics at low temperatures. [88] Other scholars have studied the preparation of polyurethane foam with natural materials as raw materials and studied the performance changes after low-temperature impact. The results show that higher mechanical strength is obtained at low temperatures [107,108]. Polyurethane foam is inevitably affected by impact load when it is in service at low temperatures. Scholars have studied the mechanical response of polyurethane foam under different strain rate impacts at low temperatures. With the decrease in temperature, due to the increase in impact resistance, the load distribution of polyurethane foam is uniform, which leads to the increase in elasticity, specific elasticity, absorption energy, and specific absorption energy [109].

The researchers also characterized the dynamic mechanical properties of glass fiber-reinforced polyurethane foam at low temperatures. The properties of glass fiber-reinforced polyurethane foam materials with different densities and orientations were studied by quasi-static, dynamic mechanical analysis (DMA), and low-velocity impact tests. The results show that the yield strength of polyurethane foam increases with the increase in loading speed at both room temperature and low temperature. The failure mode of the material is independent of the strain rate, but it is related to the temperature, and it shows more obvious brittle characteristics at low temperatures [110].

The polyurethane foam material used for thermal insulation in roof or pipeline transportation is in the stress field of temperature and humidity coupling for a long time. The change in temperature and humidity will cause the hydrolysis of the urethane group of RPUF insulation material, resulting in material aging [111], resulting in the destruction of cell structure and the decrease in thermal insulation performance of RPUF. Under the action of multi-field coupling, the existence of additional stress harms the thermal conductivity of RPUF. RPUF is more prone to aging, and the thermal insulation performance decreases [112,113].

## 4. Conclusions and Discussion

Polyurethane foam materials have been widely used in many fields because of their excellent properties. Due to their good expansion and self-adaptive properties and good compressive and tensile strength, they play an important role in engineering fields such as impermeability repair, ground lifting, road maintenance, and foundation reinforcement. Polyurethane foam materials are temperature-sensitive materials, and this paper reviews the existing research at home and abroad from the point of view of the influence of temperature on polyurethane foam properties during the polyurethane foams casting and molding stages and in service. The conclusions are as follows.

(1) Temperature affects the strength of polyurethane foams in both the casting and molding stages as well as in the service stage, and polyurethane foams materials generally show a significant reduction in strength as the temperature increases. However, the difference between the two stages is that changing the mold temperature, the raw material temperature, or the ambient temperature at the time of casting has an effect on the cell structure of polyurethane foams in the casting and molding stage. While changing the temperature in the service stage only changes the strength of the material, there is no significant change to its cell structure.

(2) Since the polymer foaming stage is mainly a chemical exothermic reaction, the –NCO bond of polyisocyanate and the –OH bond of polyether polyol can fully react at a higher temperature, releasing a large amount of heat and forming more cells, which leads to the increase in porosity and lower density after polyurethane foams molding. Furthermore, because the low temperature can remove the reaction heat faster, it also leads to a small polyurethane foam foaming multiple, a small cell size, and a high density of finished products. However, there is no systematic study on the mechanism of the molecular action of polyurethane foams affected by temperature in the service stage, and whether the change in its mechanical properties depends on the change in microstructure is still an issue worthy of study and discussion.

(3) At present, most of the studies on the influence of temperature on the mechanical properties of polyurethane foam materials during the service stage are at the macroscopic level, and there are fewer studies on the mechanism of its action and the connection between the microstructure and the change in the properties of the matrix material. Therefore, it is the focus of future research to study the mesostructure changes in polyurethane foams affected by temperature and to link the fine structure with numerical simulation and macroscopic mechanical tests.

(4) In recent years, the application of polyurethane-modified composites in engineering has become more and more widespread [114,115], and the influence of temperature on composites is more complex compared with pure polyurethane foam materials. Moreover, the study of the performance of composites under the influence of temperature and the mechanism of action is of certain significance for engineering construction.

## Figures and Tables

**Table 1:** Prediction model of polyurethane foams strength with respect to temperature.

Refer	Method	Model
Jeong-Ho Lee [72]	Modification of Two Existing Material Models Originally Applied to Metals (i.e., KHL and GTNL)	Strain-Rate and Temperature-Dependent Elasto-Viscoplastic Damage Model for Polymer Foam
Jia Li [73], Zhang Jingwei [74]	Generalized Maxwell Model (GMM)	Viscoelastic Ontological Model for Non-Water Reactive Polyurethane Grouting Materials
Ivan Kopal [75]	Artificial neural network model	Temperature-dependent model of dynamic mechanical properties and viscoelastic behavior of polyurethanes over a wide temperature range
Bipul Barua [76]	Tensile index (SE) model	Stress relaxation model for polyurethanes considering density and temperature
Dan [77]	Nagy-type image-only model for reconstruction of engineering stress-strain curves	Modeling the effect of temperature and test rate on the specific strain energy absorbed by polyurethane materials
Richeton [78]	Combining the physical frequency/strain rate dependence of the energy storage modulus and the initial Young’s modulus to improve the Weibull statistical model	Physically based robust model for predicting stiffness moduli for various temperatures and frequency/strain rates
Michael K. Neilsen [79]	/-	Models describing the effects of load path, strain rate and temperature on mechanical response

## Data Availability

Not applicable.
